# The role of bipolar radiofrequency in the treatment of vaginal laxity: a single-center cohort study in premenopausal women

**DOI:** 10.1007/s10103-026-05010-3

**Published:** 2026-09-01

**Authors:** Pablo González Isaza, Diana Velez, Manuela Cerón, Mary Ríos, Diana Carolina Bermúdez, Jahir Alexander Soto-Mora

**Affiliations:** 1 Soranus Center for Aesthetic and Functional Urogynecology, Pereira, Colombia; 2 Clinical Research Unit, ESE University Hospital San Jorge Pereira, Pereira, Colombia; 3 ODDS Epidemiology, Bogota, Colombia

**Keywords:** Vaginal laxity, Bipolar radiofrequency, Pelvic floor, Sexual function, Observational study

## Abstract

Vaginal laxity is a prevalent condition that can impair sexual function. Bipolar radiofrequency (RF) is a minimally invasive treatment; however, prospective evidence remains limited. This study aimed to evaluate changes in both subjective and objective measures of vaginal laxity following bipolar RF. This prospective observational study enrolled premenopausal women with symptomatic vaginal laxity who received four intravaginal bipolar RF sessions. The primary outcome was the change from baseline to 6 months in the Vaginal Laxity Questionnaire score. The secondary outcomes included change in genital hiatus area assessed by transperineal ultrasound, vaginal flatus frequency, vaginal laxity bother, sexual function, sexual distress, and patient-reported global improvement. Analyses were performed using paired Student t tests for genital hiatus and Friedman tests for repeated measures for patient-reported outcomes, with corresponding effect sizes reported. Thirty-two women completed follow-up. VLQ scores increased from a median of 2.0 at baseline to 4.0 at 6 months (*p* < 0.001). Regarding the secondary objective outcome, mean GH area decreased from 21.4 cm^2^ to 18.3 cm^2^ (*p* < 0.001; Cohen’s d = 0.89). FSFI scores increased, and FSDS-R scores decreased over time (both *p* < 0.001). LVB scores also decreased significantly (*p* < 0.001). At 6 months, 87.5% of participants reported global improvement, and no adverse events were observed. In this uncontrolled observational cohort, changes over time were observed in both subjective and objective measures of vaginal laxity in premenopausal women following bipolar RF; however, given the absence of a control or sham group, a contribution of placebo effect or expectation bias to the observed outcomes cannot be excluded.

## Introduction

Vaginal laxity (VL) is a prevalent symptom of pelvic floor dysfunction, characterized by the perceived loss of vaginal tone and elasticity [1]. It significantly impacts quality of life and sexual function, with a reported prevalence of 24–35% [2], yet it remains a frequently underreported condition [1], Its symptomatic spectrum extends beyond sexual impact and can include vaginal flatulence and mild urinary incontinence [1], further complicating its management.

The etiology of VL is multifactorial; vaginal childbirth is the primary risk factor, causing trauma and overdistension of the pelvic floor musculature and the genital hiatus. These acute changes are compounded by the chronic effects of aging and estrogen decline, which lead to thinning of the vaginal epithelium, reduced lubrication, and alterations in connective tissue [2]. Consequently, the management of VL has evolved to include a spectrum of options, from conservative pelvic floor therapy to surgical interventions such as perineoplasty [3]. In recent years, minimally invasive energy-based devices, particularly radiofrequency (RF) systems, have gained popularity as effective, non-surgical alternatives, a trend supported by studies reporting high patient satisfaction and improvements in sexual function [4, 5]. The proposed mechanisms of RF involve the delivery of controlled thermal energy (40–45 °C) to the vaginal tissue. It has been suggested that this heat may induce a wound‑healing response, with fibroblast activation and subsequent collagen and elastin remodeling, potentially contributing to changes in vaginal tone and elasticity.

Vaginal laxity syndrome (VLS) is increasingly recognized as a distinct clinical entity within the spectrum of pelvic floor dysfunction. A 2023 scoping review of available measurement tools for vaginal laxity identified substantial heterogeneity in assessment approaches, with most studies relying on subjective patient-reported measures and few incorporating objective anatomical endpoints [6]. This reflects a broader pattern documented in the literature: a recent systematic review of patient-reported outcomes and measures used in VLS research found that 68 distinct outcomes and 57 different instruments have been used across 69 studies, with the FSFI (47.8%) and VLQ (24.3%) among the most frequently employed, yet 81% of tools remain unvalidated [7]. A parallel systematic review similarly identified measurement heterogeneity as a critical methodological challenge in the field, further supporting the need for standardized, objective endpoints [8]. These findings underscore the importance of combining validated patient-reported measures with objective anatomical assessment, as undertaken in the present study.

Among RF modalities, the bipolar configuration offers a potential safety advantage by delivering more focused, superficial energy, which may improve patient tolerance. However, clinical evidence supporting its use specifically for vaginal laxity requires strengthening. While systematic reviews confirm the symptomatic efficacy of energy-based treatments, they concurrently highlight a critical reliance on subjective endpoints and a lack of data with objective anatomical measures [9]. This gap is clinically significant: a recent randomized sham-controlled trial of a transcutaneous RF device demonstrated notable subjective improvement but found no significant change in genital hiatus measurement [10], underscoring the uncertainty regarding whether RF is associated with measurable structural change. Furthermore, prospective studies of other RF configurations report positive outcomes [11]. Evidence for a dedicated bipolar RF device, particularly one incorporating validated sonographic anatomical assessment, remains lacking.

Therefore, this prospective study aimed to describe changes in clinical and functional outcomes following bipolar radiofrequency in premenopausal women with symptomatic vaginal laxity, integrating validated patient-reported measures and objective sonographic assessments over a six-month follow-up period.

## Methods

### Study design and setting

This was a prospective, single-center, observational cohort study conducted in a specialized urogynecology unit in Pereira, Colombia. The study was carried out within a routine clinical practice setting, where no interventions were assigned by the investigators, and all treatments were delivered as part of standard care.

#### Clinical trial number

not applicable.

### Inclusion and exclusion criteria

Premenopausal women aged 21–52 years presenting with symptomatic vaginal laxity were consecutively enrolled in 2025. Vaginal laxity was confirmed using the Vaginal Laxity Questionnaire, with eligible participants scoring between 1 and 3. All participants had a history of at least one term vaginal delivery (> 36 weeks), with the most recent delivery occurring more than 12 months before enrollment and reported being sexually active. Additionally, none of the patients had previously undergone or were concurrently undergoing pelvic floor physiotherapy.

Key exclusion criteria included a genital hiatus area > 26 cm^2^ on baseline transperineal ultrasound, pelvic organ prolapse beyond the hymenal plane, active genital infection or dermatosis, chronic vulvovaginal pain syndromes, prior treatment with energy-based vaginal devices within the previous 12 months, and the use of medications or conditions known to significantly affect sexual function.

### Sample size

The sample size calculation was based on a longitudinal repeated-measures study design with within-subject comparisons, considering as primary outcome perceived vaginal laxity evaluated using the Vaginal Laxity Questionnaire (VLQ), and as a secondary outcome the genital hiatus area assessed by transperineal ultrasound. For the genital hiatus outcome, the calculation was based on a paired pre - post comparison, assuming a clinically meaningful change of 3 cm^2^ and a standard deviation of the differences of 6 cm^2^. These assumptions were based on clinical experience, as no prior studies reporting this measurement before and after non-surgical interventions in this population were identified. Under these assumptions, with a significance level of 0.05 and 80% power, and applying the relative efficiency correction of the Wilcoxon test, the required sample size was estimated at 33 participants.

For the VLQ score, the calculation was based on a repeated-measures design with four time points (baseline, 1, 3, and 6 months), assuming pairwise within-subject comparisons using the Wilcoxon signed-rank test. A minimal clinically important difference of 1 point and a standard deviation of 1.35 were assumed, based on data reported by Wattanakrai et al. [12]. As six pairwise comparisons were planned (baseline vs. 1 month, baseline vs. 3 months, baseline vs. 6 months, 1 vs. 3 months, 1 vs. 6 months, and 3 vs. 6 months), a Bonferroni correction was applied, resulting in an adjusted significance level of α = 0.0083. Under these assumptions, the estimated sample size was 27 participants to achieve 80% power. The final sample size was 34 to account for potential loss to follow-up.

### Standard treatment

Participants received intravaginal bipolar radiofrequency as part of routine clinical care following the institutional protocol. The treatment consisted of four sessions administered at approximately two-week intervals by a certified gynecologist using an approved device (InMode Ltd., Toronto, Canada).

The system used was the InMode Votiva with a disposable FormaV intravaginal handpiece, which delivers bipolar RF energy at a target temperature of 40–45 °C monitored in real time by an integrated thermocouple. Each session consisted of three passes (anterior, posterior, and lateral walls) lasting 15–20 min, with power automatically adjusted to maintain the target temperature. Before the procedure, the external genitalia were cleansed with an antiseptic solution, and a non‑sterile ultrasound gel was applied as a coupling agent; no local anesthesia was required. After each session, patients were advised to refrain from sexual intercourse, vaginal douching, or strenuous physical activity for 24 h. No modifications to the intervention, device, or clinical indication were introduced for study purposes.

### Data collection and measures

Clinical assessments were conducted as part of routine clinical care at baseline (pre-treatment) and at 1, 3, and 6 months following the final treatment session. Genital hiatus (GH) area was assessed by one of the authors using transperineal ultrasound while the patient was in resting position (measured only at baseline and 6 months). Patient-reported outcomes collected were the Vaginal Laxity Questionnaire (VLQ), a 7-point Likert scale ranging from 1 (very loose) to 7 (very tight), where higher scores indicate greater perceived vaginal tightness [13]; vaginal flatus (VF) frequency, measured using an ordinal Likert scale from 1 (rarely) to 5 (almost always); the Vaginal Laxity Bother (LVB) score [14]; a 10-point Likert scale ranging from 0 (no bother) to 10 (maximum bother), reflecting the degree of discomfort associated with vaginal laxity [14]; and the Global Rating of Improvement (GRA), a Likert-type scale assessing the patient’s perceived overall change following treatment [13]. Sexual function was evaluated using the Female Sexual Function Index (FSFI) [15], a validated multidimensional instrument with a total score ranging from 2 to 36, where higher scores indicate better sexual function, and sexually related distress was assessed using the Female Sexual Distress Scale-Revised (FSDS-R) [16], with total scores ranging from 0 to 52, where higher scores indicate greater distress. The FSFI and VLQ were selected as primary patient-reported measures given their status as the most frequently used and best-validated instruments in VLS research [6–8]. Adverse events were defined as any untoward medical occurrence and were actively queried at each follow-up visit, combined with a focused gynecological examination.

### Ethics

The study protocol was approved by an independent ethics committee (Connectica SAS Ethics Committee; Approval Minute AC-CNA-04 Protocol 003CON-PG-2025). The study was conducted in accordance with the Declaration of Helsinki and local regulatory requirements.

### Human ethics and consent to participate declaration

All participants provided written informed consent prior to enrollment in the study.

### Statistical analysis

Descriptive statistics were used to summarize the data. Continuous variables with a normal distribution are presented as mean (SD), whereas non normally distributed continuous variables are reported as median (interquartile range [IQR]). Categorical variables are expressed as counts (n) and percentages (%). Normality was assessed using the Shapiro-Wilk test and Q-Q plots.

The primary outcome was the change from baseline to the 6-month visit in VLQ score. Secondary outcomes included change in GH area measured by transperineal ultrasound, and patient-reported measures assessed longitudinally during follow-up. Changes in GH measurements were analyzed using paired Student’s t tests. Patient-reported outcome measures, including VLQ, VF, LVB, FSFI, and FSDS-R, were analyzed using the Friedman test for repeated measures. When statistically significant, pairwise comparisons were performed using the Conover post hoc test with adjustment for multiple comparisons. Bonferroni correction was applied to account for multiple pairwise comparisons across time points.

Likert-scale data were treated as ordinal and summarized using medians and interquartile ranges, with non-parametric methods applied for analysis, in line with recommendations for ordinal data analysis [17, 18].

Effect sizes were estimated using Kendall’s W for Friedman tests and Cohen’s d for paired Student’s t tests. GRA at 6 months and baseline POP - Q measures are presented descriptively. All analyses were performed using R software, and a P value < 0.05 was considered statistically significant.

## Results

A total of 34 premenopausal women with symptomatic vaginal laxity and at least one prior term vaginal delivery were enrolled and underwent bipolar radiofrequency. Complete follow-up data was available for 32 participants, who comprised the final analytic sample. The mean (SD) age was 38.9 (6.1) years, and the mean (SD) body mass index was 24.9 (3.3). The number of deliveries per patient ranged from 1 to 4, with most participants having had one (53.1%) or two (31.3%) prior vaginal deliveries and only 1 patient having had 4 previous vaginal deliveries. 84.4% of patients were classified as POP-Q stage 0. Baseline pelvic floor strength was assessed using the Oxford scale, with scores ranging from 2 to 4, a mean score of 2.53, and most participants having a score of 2, indicating weak, barely perceptible muscle contraction. This is consistent with the expected profile of women presenting with symptomatic vaginal laxity following childbirth, in whom some degree of pelvic floor muscle weakness is a common finding. No adverse events were observed during the study period.

Two participants did not complete the six-month follow-up (retention rate: 94.1%),one due to contracting an urinary tract infection, and the other one stopped attending the sessions for unknown reasons. Both were parous women aged 33 and 41 years, with a BMI of 22 kg/m² and an Oxford scale score of 2, values consistent with the range observed in the completing sample. Baseline scores on the VLQ, VLB, and vaginal flatulence items were also within the range of the completing cohort. A degree of heterogeneity was noted in patient-reported sexual function and distress: one participant had a baseline FSFI score of 18.7 and an FSDS-R score of 2, while the other scored 30.0 and 9, respectively, reflecting the variability present across the broader sample at baseline. Given the low attrition rate and the overall comparability of these participants’ baseline profiles with those of the completing cohort, systematic attrition bias is considered unlikely, though the small number of withdrawals precludes formal statistical assessment.

**Fig. 1:**
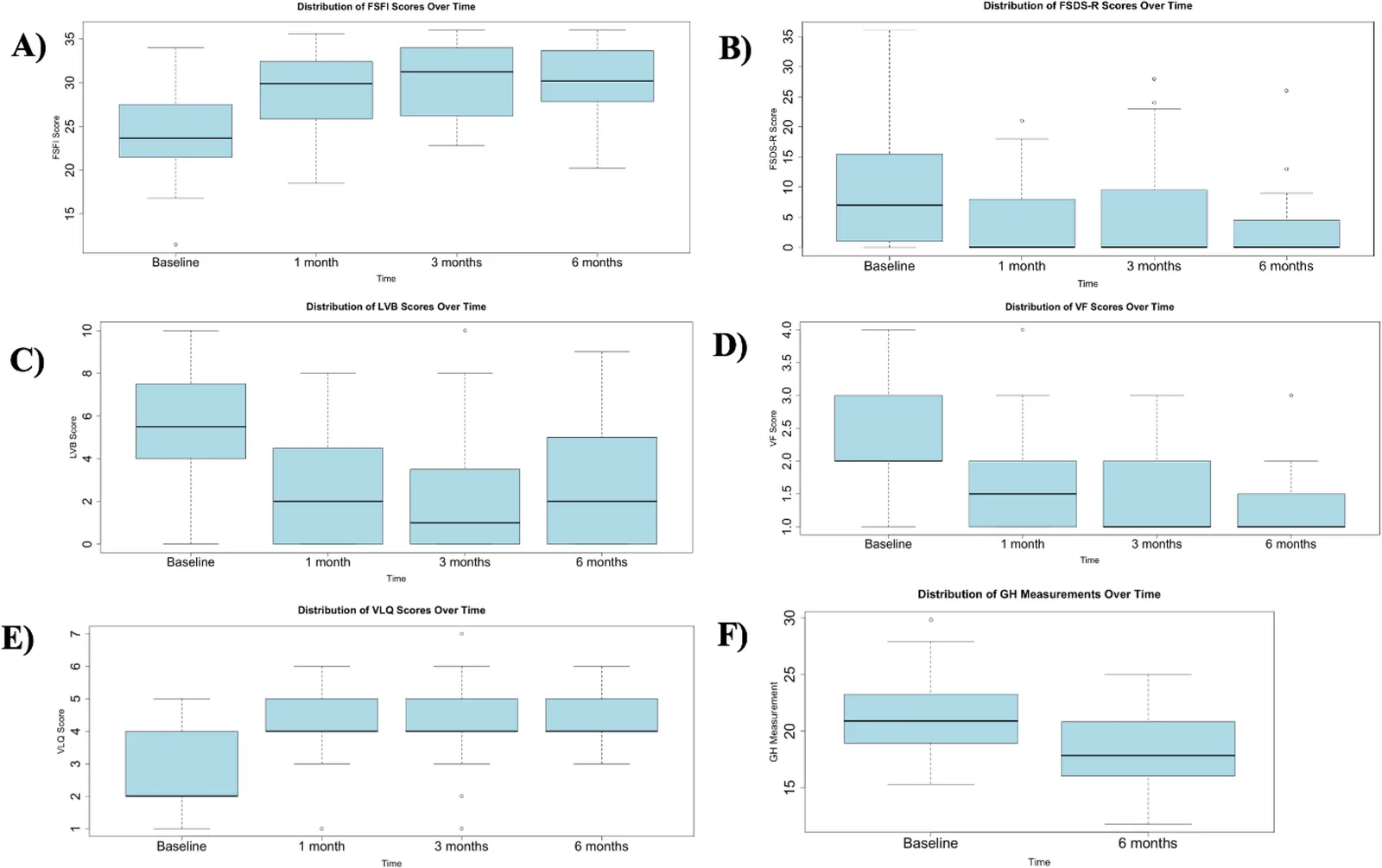
Boxplot distribution of clinical and functional outcomes at different time points FSFI: Female Sexual Function Index; FSDS-R: Female Sexual Distress Scale-Revised; LVB: Vaginal Laxity Bother; VF: Vaginal Flatus; GH: Genital Hiatus. (**A**) Female sexual function index; (**B**) Female fexual distress scale–revised; (**C**) Vaginal laxity–bother; (**D**) Vaginal flatus; (**E**) Vaginal laxity questionnaire; (**F**) Genital hiatus area

Female sexual function, assessed using FSFI, differed significantly across follow-up time points (*p* < 0.001). Median FSFI scores increased from 23.6 at baseline to 29.9 at 1 month, 31.3 at 3 months, and 30.2 at 6 months (Fig. 1). Pairwise comparisons showed significant differences between baseline and all follow-up assessments, with no significant differences among post treatment time points. The effect size was moderate (Kendall’s W = 0.31) (Table 1).

**Table 1:** Changes in clinical and functional outcomes over time

Outcome	Baseline	1 month	3 months	6 months	Test statistic	Effect size	*p*-value	
Vaginal Flatus Frequency, median (IQR)	2.00 (2.00–3.00)	1.50** (1.00–2.00)	1.00** (1.00–2.00)	1.00** (1.00–1.25)	χ²(3) = 33.1	0.34^c^	<.001^d^	
Perceived Vaginal Laxity, median (IQR)	2.00 (2.00–4.00)	4.00** (4.00–5.00)	4.00** (4.00–5.00)	4.00** (4.00–5.00)	χ²(3) = 48.8	0.50^c^	<.001^d^	
Female Sexual Function, median (IQR)	23.6 (21.5–27.3)	29.9** (26.0–32.3)	31.3** (26.3–33.9)	30.2** (27.9–33.6)	χ²(3) = 29.9	0.31^c^	<.001^d^	
Sexual Distress, median (IQR)	7.00 (1.00–15.3)	0.00** (0.00–7.00)	0.00** (0.00–9.25)	0.00** (0.00–4.25)	χ²(3) = 18.1	0.18^c^	<.001^d^	
Vaginal Laxity Bother, median^,^ (IQR)	5.5 (4.00–7.25)	2.00** (0.00–4.25)	1.00** (0.00–3.25)	2.00^**^ (0.00–5.00)	χ²(3) = 29.5	0.30 ^c^	< 0.001 ^d^	
Genital Hiatus, mean (± SD)	21.4 (3.17)	●	●	18.3 (3.32)	t(31) = −5.10	0.89^f^	<.001^e^	

Test statistics correspond to Friedman χ², repeated-measures ANOVA F values, or paired t-test statistics.

**Significant change when conover post hoc pairwise comparisons were applied between baseline and each follow-up time point (*P* < 0.05)

^c^ Kendall’s W

^d^ P values correspond to the overall comparison across time points assessed using the Friedman test

^e^Pairwise comparisons between two points were analyzed using paired Student t test

^f^Cohen’s d

A similar pattern was observed for sexual distress (FSDS-R), which also changed significantly over time (*p* < 0.001). Median scores decreased from 7.0 at baseline to 0.0 at 1 month and remained unchanged at 3 and 6 months. Post hoc comparisons indicated significant differences between baseline and all follow-up assessments (Fig. 1). No differences were observed among post-treatment time points. The effect size was small (Kendall’s W = 0.18) (Table 1).

VLB scores differed significantly across time points (*p* < 0.001). Median scores decreased from 5.50 at baseline to 2.00 at 1 month, 1.00 at 3 months, and a slight increase back to 2.00 at 6 months. Post hoc pairwise comparisons showed significant reductions from baseline to all follow-up time points, with no significant differences among post-treatment assessments. The overall effect size was small to moderate (Kendall’s W = 0.30) (Table 1).

VF frequency demonstrated an early decrease that was maintained over time. Median VF scores decreased from 2.0 at baseline to 1.5 at 1 month and to 1.0 at both 3 and 6 months (Fig. 1). Overall differences across time points were statistically significant (*p* < 0.001), with a moderate effect size (Kendall’s W = 0.35) (Table 1).

The primary outcome, perceived vaginal laxity assessed through the VLQ, differed significantly over time (*p* < 0.001). Median scores increased from 2.00 at baseline to 4.00 in the 1-, 3-and 6-month follow-ups. Pairwise analyses indicated that all post-treatment assessments differed significantly from baseline, with no differences among follow-up visits (Fig. 1). The magnitude of change was large (Kendall’s W = 0.51) (Table 1).

Mean GH area decreased from 21.4 cm^2^ at baseline to 18.3 cm^2^at 6 months (mean difference: − 3.11 cm^2^; 95% CI: − 4.36 to − 1.86; *p* < 0.001) (Fig. 1), with a large effect size (Cohen’s d = 0.89) (Table 1), alongside changes in patient reported outcomes.

Global perception of change at 6 months, assessed using the GRA scale, indicated a high rate of perceived improvement following treatment. At the 6-month follow-up, 28 participants (87.5%) reported improvement (scores 5–7), while 4 (12.5%) reported no change (score 4); no participants reported worsening of symptoms (scores 1–3). Finally, no adverse events or treatment-related complications were observed.

## Discussion

This prospective observational study described changes over time in both subjective and objective measures of vaginal laxity in premenopausal women following bipolar radiofrequency. In the absence of a control or sham group, these changes cannot be attributed to the intervention, and the contribution of placebo effect or expectation bias cannot be excluded. While systematic reviews have noted the scarcity of prospective data incorporating objective anatomical endpoints [9, 19], recent evidence further emphasizes this gap.

A 2025 systematic review and meta-analysis of energy-based device therapy for VLS found that improvements in FSFI and VLQ scores in observational studies were consistent with our findings, though the same review noted that controlled trials did not confirm statistically significant benefits compared to sham, underscoring the limitations inherent in uncontrolled designs such as ours [20]. A 2025 narrative review of RF therapy in pelvic floor disorders similarly concluded that the evidence base for RF in VLS is still exploratory, characterized by small sample sizes, single-center designs, and short follow-up periods which are limitations shared by the present study [21]. Our findings help address this gap by including both sonographic GH area measurements and validated patient-reported outcomes. Specifically, we observed a sustained reduction in GH area over six months, accompanied by changes in patient-reported laxity (VLQ), with a large effect size. The simultaneous documentation of objective GH reduction and subjective laxity relief provides evidence that addresses the previously identified lack of combined objective-subjective data in this field.

The observed changes are consistent with the proposed thermal induction of neocollagenesis and neoelastogenesis. While we did not perform histological analysis, the significant and sustained objective reduction in GH area aligns with this model. Interestingly, a prior pilot study using radiofrequency found significant symptomatic improvement without a concurrent increase in vaginal pressure measurements [22], suggesting that the clinical perception of “tightening” is multifactorial. Our findings of anatomical changes alongside a reduction in symptom bother and sexual distress may reflect a combination of structural support and mucosal or neurosensory contributions. This is further supported by the improvements across all domains of the Female Sexual Function Index and the reduction in sexual distress (FSDS-R), outcomes also reported in other studies of radiofrequency and laser therapies [12, 19, 23].

The objective reduction in GH area is a noteworthy finding. This anatomical change is consistent with previously documented associations between radiofrequency and pelvic floor support structures, including improvements in urethral hypermobility assessed via Q-tip test following transvaginal RF treatments for stress urinary incontinence [24, 25]. However, direct comparisons with SUI studies should be interpreted with caution as our study population was not selected for urinary symptoms, and no urodynamic or urinary outcome data were collected. The observed GH reduction therefore cannot be attributed to the same mechanisms underlying RF effects in SUI, and any such parallel remains speculative. Nonetheless, these convergent findings may be consistent with the broader hypothesis that RF energy influences connective tissue and muscular support across the distal pelvic floor, warranting further future research.

The secondary outcomes provide a comprehensive, objective and subjective, view of the changes observed following treatment. Among the patient-reported secondary outcomes, the reduction in the frequency of vaginal flatus, an underreported symptom, underscores functional changes at the vaginal introitus. The early and sustained changes in all measured outcomes, with most differences evident by the one-month follow-up and maintained at six months, suggest a durable association. This stability is similar to findings from other studies, where benefits plateaued after an initial post-treatment period [22, 23]. Importantly, the high rate of global perceived improvement (87.5%) and the absence of any adverse events are consistent with the safety and tolerability profile reported for radiofrequency-based treatments [12, 19].

These collective findings are consistent with a broad positive pattern of change across multiple domains of vaginal laxity, spanning objectively measured structure (GH), patient-reported sensation and distress, and specific functional symptoms. These results are promising, however, given the observational design and absence of a comparator group, caution is warranted in interpreting these findings. The observed changes may reflect, at least in part, placebo effect, expectation bias, or natural history, and while they could indicate the efficacy of treatment, they are not definite evidence of treatment efficacy and need to be confirmed by evidence from controlled trials.

When contextualizing our results within the existing evidence, they are consistent with the conclusions of recent reviews and trials. Our data aligned with the findings of a sham-controlled randomized trial that also reported histological evidence of tissue regeneration [12]. These findings are also consistent with the pooled estimates reported in the most recent systematic review and meta-analysis of energy-based devices for VLS, which found significantly higher FSFI and VLQ scores after RF treatment in observational studies [20].Furthermore, our use of a bipolar device adds to the evidence base for this specific configuration, which may have a different safety profile due to more controlled energy penetration. The changes in sexual function scores we observed are a consistent theme across the literature for both laser and other RF devices [19, 23].

## Limitations and strengths

This study has several limitations. First, as a single-center observational cohort without a control or sham group, the observed changes cannot be attributed exclusively to the intervention. Second, the sample size was relatively small and derived from a specialized clinical setting, which may limit external validity and generalizability to broader populations. Third, the objective measures were not collected by a blinded clinician. Fourth, although a prospective design was implemented, the follow-up period of six months may be insufficient to evaluate long-term durability of treatment effects or the potential need for maintenance therapy. Fourth, residual confounding cannot be excluded, as factors such as age, parity, and baseline symptom severity may be associated with the observed outcomes. Finally, the study population was restricted to premenopausal women, which may limit applicability to postmenopausal populations with different underlying tissue characteristics.

Additionally, the use of non-standardised assessment protocols remains a recognized challenge in the field. Recent systematic reviews have highlighted that the majority of PROMs used in VLS research are unvalidated and applied inconsistently across studies, complicating cross-study comparisons [7, 8]. The present study addressed this partially by selecting the FSFI and VLQ — the two most commonly used and accepted measures — alongside objective GH measurement; however, further standardization of outcome sets, ideally through consensus-based core outcome sets currently under development for VRS [7], will be essential for future work.

Despite these limitations, this study also has several strengths. The prospective design enabled a longitudinal assessment of outcomes within a routine clinical practice setting, capturing changes over time under real-world conditions. The combined use of objective measures, such as GH assessed by transperineal ultrasound, and multiple patient-reported outcomes provided a comprehensive assessment across anatomical, functional, and patient-centered domains. In addition, consecutive patient enrollment may help minimize selection bias and support the representativeness of the study population. The use of validated instruments further contributes to the robustness of the findings.

## Conclusions

In conclusion, this prospective observational study described changes over time in both subjective and objective measures of vaginal laxity in premenopausal women following bipolar radiofrequency, with no treatment-related adverse events observed. However, as an uncontrolled observational cohort without a sham or comparator group, the observed changes cannot be attributed exclusively to the intervention, and a contribution of placebo effect or expectation bias cannot be excluded. Additionally, the limited follow-up time and sample size warrant caution in relation to the safety of the treatment, which cannot be unequivocally asserted. These findings contribute to the growing body of real-world evidence in this field and may be consistent with a potential role for this modality in the management of symptomatic vaginal laxity. Controlled studies with longer follow-up are warranted to establish efficacy, define long-term outcomes, and determine optimal treatment strategies.

## Data Availability

The data that support the findings of this study were obtained from patient medical records. Due to privacy and confidentiality restrictions, these data are not publicly available. Data may be shared upon reasonable request and with authorization from the participating clinical center and in accordance with applicable ethical and institutional regulations.

## References

[1] Fait T, Baltazár T, Bubenickova L (2023) Treatment of vulvovaginal laxity by electroporation: the jett plasma medical for her II Study. J Clin Med 12(19):6234–6234. 10.3390/JCM12196234 37834879 PMC10573526

[2] Talab S, Al-Badr A, AlKusayer GM, Dawood A, Bazi T (2019) Correlates of vaginal laxity symptoms in women attending a urogynecology clinic in Saudi Arabia. Int J Gynaecol Obstet 145(3):278–82. 10.1002/IJGO.12810 30924525 PMC7027493

[3] Sezgic M, Karakol P (2023) Vaginoplasty and perineoplasty. In: postpartum body changes. pp. 581–608. 10.1007/978-3-030-43840-1_35

[4] Ricottone J, Richards R, Reese A, Burke M, Brennan M (2024) (196) Vaginal rejuvenation by laser or radiofrequency: a systematic review of the literature. J Sex Med 21(Supplement_7). 10.1093/JSXMED/QDAE167.191

[5] Magon N, Alinsod R (2016) ThermiVa: the revolutionary technology for vulvovaginal rejuvenation and noninvasive management of female SUI. J Obstet Gynaecol India 66(4):300. 10.1007/S13224-016-0868-0 27382227 PMC4912496

[6] Aulia I, Valeria M (2023) Current perspectives in vaginal laxity measurement: a scoping review. Arch Plast Surg 50(05):452–62. 10.1055/a-2113-3202 37808327 PMC10556325

[7] Chen H, Meng J, Li Q, Luo X, Wang Y, Chen Y et al (2025) Patient-reported outcomes and measures for vaginal relaxation syndrome management: a systematic review. Health Qual Life Outcomes 23(1):98. 10.1186/s12955-025-02432-5 41063182 PMC12505649

[8] Chen H, Meng J, Li Q, Wang Y, Chen Y, Niu X et al (2026) Clinical outcomes and measures for vaginal relaxation syndrome: a systematic review. Value Health 29(1):149–158. 10.1016/j.jval.2025.08.012 40902796

[9] Pereira GMV, Cartwright R, Juliato CRT, Domoney C, Iglesia CB, Brito LGO (2024) Treatment of women with vaginal laxity: systematic review with meta-analysis. J Sex Med 21(5):430–442. 10.1093/JSXMED/QDAE028 38508858

[10] Solanki SB, Mishra V, Mishra N, Desai SA, Alinsod R (2025) Transcutaneous temperature-controlled radiofrequency for vaginal rejuvenation. J -Life Health 15(4):250. 10.4103/JMH.JMH_32_24 PMC11824938 39959723

[11] Fu L, Long S, Li Q (2023) The efficacy and safety of temperature controlled dual-mode radiofrequency in women with vaginal laxity. BMC Womens Health 23(1):121. 10.1186/S12905-023-02261-Y 36959573 PMC10035145

[12] Wattanakrai P, Limpjaroenviriyakul N, Thongtan D, Wattanayingcharoenchai R, Manonai J (2022) The efficacy and safety of a combined multipolar radiofrequency with pulsed electromagnetic field technology for the treatment of vaginal laxity: a double-blinded, randomized, sham-controlled trial. Lasers Med Sci 37:1829–42. 10.1007/s10103-021-03438-3 34647191 PMC8971182

[13] Millheiser LS, Pauls RN, Herbst SJ, Chen BH (2010) Radiofrequency treatment of vaginal laxity after vaginal delivery: nonsurgical vaginal tightening. J Sex Med 7(9):3088–3095. 10.1111/j.1743-6109.2010.01910.x 20584127

[14] Kaya AK, Menek MY (2026) Investigation of the effects of Kegel exercises on vaginal flatus among postpartum women: a randomized controlled trial. Ginekol Pol 97(2):95–101. 10.5603/gpl.105643 41603160

[15] Sánchez-Sánchez B, Navarro-Brazález B, Arranz-Martín B, Sánchez-Méndez Ó, de la Rosa-Díaz I, Torres-Lacomba M (2020) The female sexual function index: transcultural adaptation and psychometric validation in spanish women. Int J Environ Res Public Health 17(3). 10.3390/ijerph17030994 PMC7037847 32033334

[16] Carpenter JS, Reed SD, Guthrie KA (2015) Using an FSDS-R item to screen for sexually related distress: A MsFLASH Analysis. Sex Med 3(1):7–13. 10.1002/sm2.53 25844170 PMC4380908

[17] Jamieson S (2004) Likert scales: how to (ab)use them. Med Educ 38(12):1217–1218. 10.1111/J.1365-2929.2004.02012.X 15566531

[18] Sullivan GM, Artino AR (2013) Analyzing and interpreting data from likert-type scales. J Grad Med Educ 5(4):541. 10.4300/JGME-5-4-18 24454995 PMC3886444

[19] Juhász MLW, Korta DZ, Mesinkovska NA (2021) Vaginal rejuvenation: a retrospective review of lasers and radiofrequency devices. Dermatol Surg 47(4):489–494. 10.1097/DSS.0000000000002845 33165070

[20] Chen H, Meng J, Li Q, Pan W, Niu X, Wei D (2026) Efficacy and safety of energy-based device therapy in women diagnosed with vaginal relaxation syndrome: a systematic review and meta-analysis. Int Urogynecol J 11. 10.1007/s00192-026-06700-w 42274731

[21] Luo C, Wei D, Yu X, Chen Y (2025) Application progress of radiofrequency (RF) therapy in pelvic floor disorders (PFDs): a narrative review focusing on randomized controlled trials. Gynecol Pelvic Med 8:25–25. 10.21037/gpm-25-36 42211909 PMC13215003

[22] Kim JH, Kim K, Ahn S (2020) Pilot study of radiofrequency thermal therapy performed twice on the entire vaginal wall for vaginal laxity. Eur J Obstet Gynecol Reprod Biol 254:159–163. 10.1016/j.ejogrb.2020.09.022 33010693

[23] Lalji S, Lozanova P (2017) Evaluation of the safety and efficacy of a monopolar nonablative radiofrequency device for the improvement of vulvo-vaginal laxity and urinary incontinence. J Cosmet Dermatol 16(2):230–234. 10.1111/JOCD.12348 28556393

[24] Razzaghi M, Asghari-azghan A, Montazeri S, Razzaghi Z, Mazloomfard MM, Vafaee R (2021) Intravaginal pulsed contractile radiofrequency for stress urinary incontinence treatment; a safety study. J Lasers Med Sci 12(1):e56. 10.34172/JLMS.2021.56 35155141 PMC8837860

[25] Wang XX, Xu Y, Song YF, Zheng XH, Jiang XX, Lin CQ (2025) Efficacy of two modes of transvaginal temperature-controlled radiofrequency for female stress urinary incontinence. Int Urogynecol J 36(9):1759–1766. 10.1007/S00192-025-06065-6 39945801

